# Glycerol Uptake Is Important for L-Form Formation and Persistence in *Staphylococcus aureus*

**DOI:** 10.1371/journal.pone.0108325

**Published:** 2014-09-24

**Authors:** Jian Han, Lili He, Wanliang Shi, Xiaogang Xu, Sen Wang, Shuo Zhang, Ying Zhang

**Affiliations:** 1 Department of Pathogenic Biology, School of Basic Medical Sciences, Lanzhou University, Lanzhou, China; 2 Department of Molecular Microbiology and Immunology, Bloomberg School of Public Health, Johns Hopkins University, Baltimore, Maryland, United States of America; 3 Institute of Antibiotics, Huashan Hospital, Fudan University, Shanghai, China; 4 Department of Infectious Diseases, Huashan Hospital, Fudan University, Shanghai, China; The Scripps Research Institute and Sorrento Therapeutics, Inc., United States of America

## Abstract

*S. aureus* is a significant human pathogen and has previously been shown to form cell wall deficient forms or L-forms *in vitro* and *in vivo* during infection. Despite many previous studies on *S. aureus* L-forms, the mechanisms of L-form formation in this organism remain unknown. Here we established the L-form model in *S. aureus* and constructed a transposon mutant library to identify genes involved in L-form formation. Screening of the library for mutants defective in L-form formation identified *glpF* involved in glycerol uptake being important for L-form formation in *S. aureus.* Consistent with this observation, *glpF* was found to be highly expressed in L-form *S. aureus* but hardly expressed in normal walled form. In addition, *glpF* mutant was found to be defective in antibiotic persistence. The defect in L-form formation and antibiotic persistence of the *glpF* mutant could be complemented by the wild type *glpF* gene. These findings provide new insight into the mechanisms of L-form formation and persistence in *S. aureus* and may have implications for development of new drugs targeting persisters for improved treatment.

## Introduction

L-form bacteria refer to cell wall deficient form of bacteria that were first discovered by Emmy Klienenberger in 1935 [1]. There is a large body of literature on the bacterial L-forms due to their fascinating biology and their potential importance in latent and persistent infections [2], [3], [4], [5]. L-form bacteria do not grow in regular culture medium but require special culture conditions including rich medium, serum, cell wall inhibitors such as penicillin, osmotic protectant such as sucrose or sodium chloride and soft agar. In contrast to normal bacteria with cell wall that divide by binary fission mediated by FtsZ protein, L-form bacteria divide in a FtsZ-independent manner upon cell envelope stress under specialized conditions [6]. Thus L-form bacteria serve as a useful model to study cell division. There is recent interest in the molecular basis of L-form bacteria formation and survival [5], [6], [7], [8], [9]. Despite numerous studies, little is known about the mechanisms of L-form formation. Previous studies have mainly identified mutations in genes involved in cell wall synthesis or cell division in stable L-form bacteria being important for L-form formation [6], [9], [10], [11]. Using *E. coli* unstable L-form bacteria as a model, we recently systematically examined the molecular basis of L-form formation by microarray analysis and mutant screens and identified a network of genes and pathways involved in L-form formation or survival [7]. These include DNA repair and protection (SOS response), energy production, efflux/transporters, iron homeostasis, cell envelope stress, protein degradation such as trans-translation [7]. These pathways share significant similarity to those involved in bacterial persister and biofilm formation [7]. Despite the above progress, the molecular basis of L-form bacteria formation in other bacteria remains largely unknown.

*Staphylococcus aureus* is the leading cause of wound and nosocomial infections [12]. Methicillin-resistant *S. aureus* (MRSA) poses a significant threat in different parts of the world. *S. aureus* is known to form L-form bacteria *in vitro* [13], [14] or *in vivo* during infection [15], [16], [17] or after antibiotic treatment [18]. Clinical samples from patients suffering from MRSA infection contained L-form bacteria exhibiting typical “fried-egg” morphology [16]. There is the interesting observation that going through L-form stage with initial phenotypic resistance or persistence to beta-lactam cell wall antibiotic led to subsequent stable genetic resistance after reversion to walled normal form in *S. aureus* [13]. In addition, *S. aureus* has been demonstrated to form persisters in different studies [19], [20]. However, the molecular mechanisms of L-form bacteria formation and persistence in *S. aureus* are unclear. In this study, we constructed a transposon mutant library of *S. aureus* and performed a preliminary screen to identify mutants defective in L-form formation. We identified *glpF* involved in glycerol uptake being critical for L-form formation in *S. aureus.* In addition, we found that *glpF* is also important for persistence to antibiotics in *S. aureus*.

## Materials and Methods

### Antibiotics

Penicillin, ampicillin, chloramphenicol, erythromycin, tetracycline, and norfloxacin were obtained from Sigma-Aldrich Co., and their stock solutions were freshly prepared, filter-sterilized and used at appropriate concentrations as indicated.

### Bacterial strains and culture conditions

Bacterial strains and plasmids used in this study are listed in Table 1. All of the *S. aureus* strains except strain RN4220 were derivatives of the Newman strain. *S. aureus* strains were cultivated in tryptic soy broth (TSB) (BBL, 211768) and tryptic soy agar (TSA) (Difco, 236950) at 37°C. Chloramphenicol, tetracycline and erythromycin were used at concentrations 5, 2.5 and 10 µg/ml respectively for generating random transposon mutant library. Tetracycline was used at 5 µg/ml for complementation of the *glpF* mutant.

**Table 1:** Bacterial strains and plasmids used in this study.

Strain or plasmid	Relevant genotype and property	Source or reference
*S. aureus*		
Newman	Clinical isolate, ATCC 25904, saeS constitutively active	ATCC
*glpF* mutant	Derived from strain Newman with transposon insertion in *glpF* gene	This study
*glpF* mutant	*glpF* mutant transformed with pT181	This study
*glpF*-pT181-*glpF*	*glpF* mutant complemented with pT181 plus wild type *glpF* gene	This study
RN4220	Restriction-deficient shuttle plasmid host	ATCC
Plasmids		
pBursa	Transposon encoding plasmid	[12]
pFA545	Transposase encoding plasmid	[12]
pT181	Plasmid vector for transformation of *S. aureus*	[26]

### Construction of *S. aureus* transposon mutant library

*S. aureus* transposon mutant library was constructed as described [12]. Briefly, *S. aureus* RN4220 and Newman strain electrocompetent cells were prepared from log phase cultures grown in TSB medium. pBursa and pFA545 plasmid DNA were transformed into *S. aureus* competent cells by electroporation (voltage = 2.5 kV, resistance = 100 Ω, capacity = 25 µF) using MicroPulser Electroporation Apparatus (Bio-Rad). pFA545 plasmid DNA was transformed into *S. aureus* strain RN4220 and then was isolated from RN4220 for introduction into *S. aureus* Newman strain. pBursa was transformed into *S. aureus* Newman strain carrying pFA545. The cells were spread on TSA plates containing 2.5 µg/ml tetracycline and 5 µg/ml chloramphenicol followed by incubation at 30°C and then transferred onto a 43°C prewarmed TSA containing 10 µg/ml erythromycin and incubated at 43°C. About 6000 mutant clones were picked and cultured in 96 wells and then stored as transposon mutant library at −80°C until use.

### Induction of *S. aureus* L-form colonies

*S. aureus* was grown in brain heart infusion (BHI) broth (Becton Dickinson, BD) overnight to stationary phase. Undiluted cultures were spotted onto L-form induction media (LIM) which consisted of BHI supplemented with 1% agar (BD), 10% horse serum (Sigma), 3.5% sodium chloride, 20% sucrose, 0.125% magnesium sulfate, and 600 µg (1000 units)/ml of Penicillin G (Sigma). After the inoculum was absorbed into the agar, the plates were inverted and incubated at 33°C for 7∼10 days. The bacterial colonies were detected by inverted microscope (Nikon GM3) and the typical L-form colonies appeared as “fried egg”.

### Microscopy

*S. aureus* L-form bacteria were examined using a Nikon GM3 inverted microscope for “fried egg” colonies grown on LIM agar along with normal growth on a control medium without penicillin. The typical colonies were fixed by glutaraldehyde before being processed and examined by electron microscopy (EM). Scanning EM and transmission EM were performed with scanning electron microscope (JSM-6380Lv) and transmission electron microscope (JEM-1230), respectively, using procedures as described [7], [21].

### Library screen to identify mutants with defect in L-form colony formation

The library screening procedure was similar to that as we previously described [7]. Briefly, the mutant library consisting of 6076 transposon mutants of *S. aureus* Newman was grown in 200 µl BHI medium at 37°C overnight in 96-well plates without shaking. Stationary phase culture of the mutant library was transferred onto L-form medium LIM plates (150 mm) by a 96-well replicator. Plates were allowed to dry before being inverted and incubated at 33°C for up to 7 days before mutants were scored for defect in forming L-form colonies on LIM plates.

### Inverse PCR and DNA sequencing of PCR products from L-form mutants

Overnight cultures of L-form deficient mutants and the *S. aureus* parent strain Newman were centrifuged and bacterial cells were collected for DNA extraction. The genome DNA was isolated by using lysostaphin (Sigma), glass beads (0.1 mm), RNase solution (4 mg/ml), followed by phenol/chloroform extraction and ethanol DNA precipitation. The purified chromosomal DNA was digested by restriction enzyme *Aci*I (New England Biolabs) and DNA restriction fragments were then circularized using T4 DNA ligase (New England Biolabs). The ligated DNA (5 µl) was used as template for inverse PCR reaction in a 25 µl reaction volume with primers ermF and ermR (Table 2). The PCR cycling parameters were 10 min at 96°C, followed by 40 cycles of 30 s at 94°C, 30 s at 63°C, and 3 min at 72°C. The PCR products were subjected to DNA sequencing with primer ermF. The identity of the DNA sequences was searched in the NCBI database using the BLAST algorithm to identify the gene of interest.

**Table 2:** Oligonucleotide primers used in this study.

Primer name	Sequence	Source or reference
ermF	5′-TTTATGGTACCATTCATTTTCCTGCTTTTTC-3′	[12]
ermR	5′-AAACTGATTTTTAGTAAACAGTTGACGATATTC-3′	
16SF	5′-CGTGCTACAATGGACAATACAAA-3′	[27]
16SR	5′-ATCTACGATTACTAGCGATTCCA-3′	
glpKF	5′-TGGACAAGCTTGCTTCGAAC-3′	This study
glpKR	5′-GATGGAACCTTCAAGCGCAT-3′	This study
glpFF	5′-CTGGCGCGAAATTAGGTGTT -3′	This study
glpFR	5′-CGGACCTAAATCACGTGCTG -3′	This study
glpfF	5′- ATTGACGGATCCAACGCTTTCATATCG-3′	This study#
glpfR	5′ -CGCTAACCTGCAGCCATTGTACAAAATC-3′	This study#

# The underlined nucleotide sequences GGATCC and CTGCAG represent *Bam*HI and *Pst*I restriction sites incorporated for cloning the wild type *glpF* gene from *S. aureus* Newman into plasmid pT181 for complementation.

### Real-time reverse transcription PCR

Real-time RT-PCR was used to assess the level of expression of *glpF* transcription in *S. aureus* L-form versus classical form bacteria. Cultures of *S. aureus* strain Newman grown overnight in TSB were inoculated onto LIM and BHI media with sucrose control respectively. These plates were incubated at 33°C for 7 days. The colonies grown on LIM and BHI with sucrose were collected for RNA isolation. The culture samples were washed once with DEPC-H_2_O and centrifuged at 8,000 g at 4°C for 5 min. RNA was isolated according to manufacturer’s instruction (Sangon Biotech Co., Ltd., Shanghai). Primers corresponding to the genes of interest were designed using Primer Express software (Version 2.0, Applied Biosystems) (Table 2). Total RNA was converted to cDNA using Super-Script III First-Strand Synthesis (Takara Bio) as described by the manufacturer. The cDNA was used as template to perform real-time RT-PCR per instruction of the reagent kit SYBR Premix Ex Taq II (Takara Bio). The expression of 16S rRNA was used as the control for estimating the fold changes of genes of interest. Cycling parameters were 95°C for 30 s and followed by 40 cycles of 5 s at 95°C, 30 s at 60°C. Relative expression levels were determined by the comparative threshold cycle (△△Ct) method.

### Complementation of *S. aureus* mutants

The wild type *glpF* gene from *S. aureus* Newman was amplified by PCR using primers glpfF and glpfR (see Table 2). The PCR primers were taken from −134 bp and 99 bp upstream and downstream of the *glpF* gene and contained restriction sites *Bam*HI and *Pst*I respectively. The PCR parameters were: 94°C 15 min, followed by 35 cycles of 94°C 30 s, 55°C 30 s, and 72°C 2 min, and a final extension at 72°C for 10 min. The PCR products were digested with *Bam*HI and *Pst*I and then cloned into plasmid pT181 cut with the same enzymes. The ligation products were electroporated into *S. aureus* strain RN4220 and the positive clones were identified by restriction digestion and PCR. The recombinant plasmid pT181+*glpF* and the pT181 vector alone control were then transformed into *S. aureus glpF* mutant by electroporation as described above under Construction of *S. aureus* transposon mutant library.

For complementation of the *glpF* mutant in L-form formation, *S. aureus* Newman strain was grown in BHI broth, and both *glpF* mutant-pT181 vector control and the *glpF* pT181-glpF complemented strain were grown in BHI broth with 5 µg/ml tetracycline overnight to stationary phase. The strains were spotted onto LIM and incubated aerobically at 33°C for 5 days followed by detection of typical L-form colonies which appeared as “fried egg” by inverted microscope as described above.

### Effect of glycerol on L-form formation

The stationary phase cultures of wild type *S. aureus* Newman strain grown in BHI broth and *glpF* mutant and the complemented strain in BHI broth containing 5 µg/ml tetracycline were spotted onto LIM without glycerol and with varying concentrations (0, 0.1%, 1%, and 10%) of glycerol, respectively. After incubation aerobically at 33°C for 5 days, the “fried egg” colonies were detected to confirm the effects of glycerol on *glpF* mutant L-form formation.

### Persister assays

For antibiotic exposure, *S. aureus glpF* mutant, the *glpF* mutant complemented strain, and *S. aureus* parent strain Newman were grown to stationary phase overnight when antibiotics ampicillin (50 µg/ml) and norfloxacin (40 µg/ml) were added to undiluted cultures and incubated without shaking for various times up to 7 or 8 days. Aliquots of bacterial cultures exposed to antibiotics were taken at different time points and washed and then plated onto TSA plates for CFU counting.

## Results and Discussion

### Generation of antibiotic induced unstable L-forms of *S. aureus*

**Figure 1:**
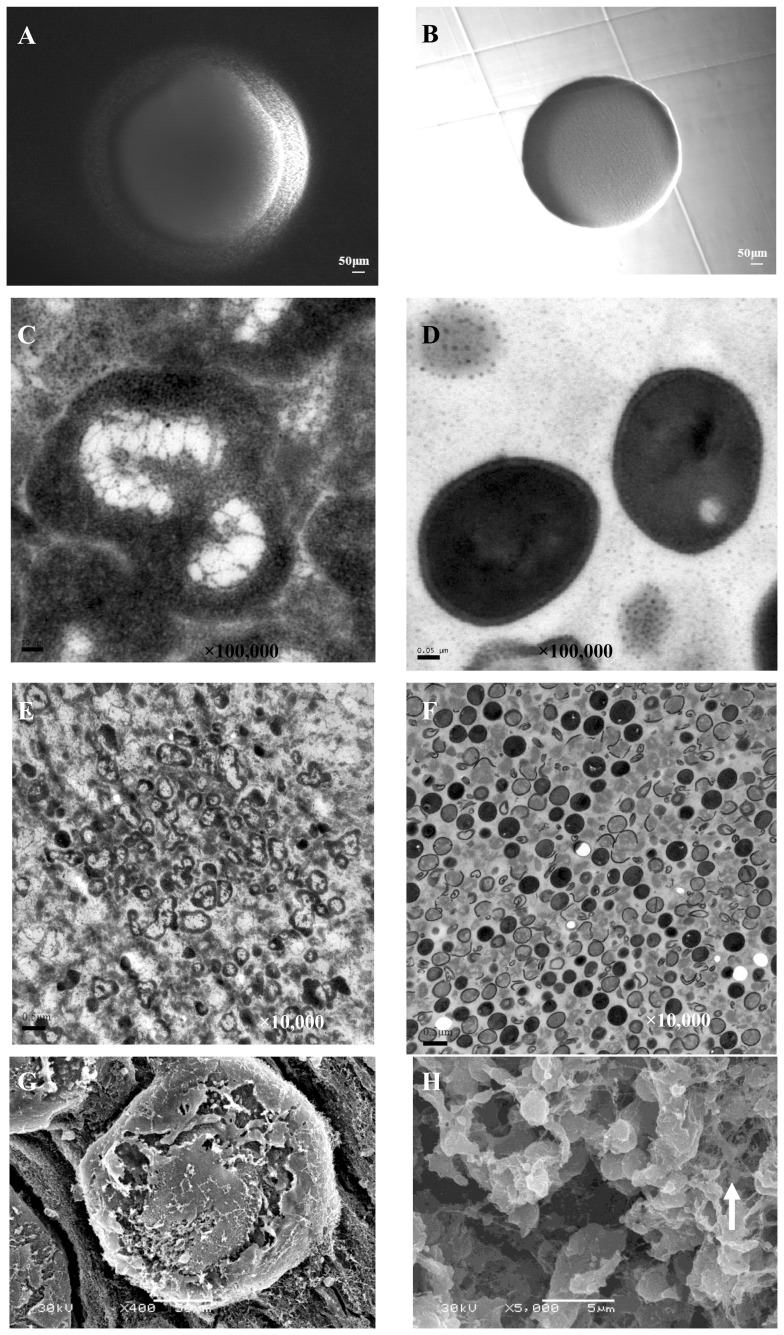
Comparison of L-form and classical form *S. aureus* morphologies. (A). *S. aureus* Newman L-form colony on L-form induction media (LIM) exhibiting typical “fried egg” morphology. (B). Control classical *S. aureus* colony on BHI medium with sucrose control but without penicillin. (C). *S. aureus* L-form shape and structure (TEM, ×100,000). *S. aureus* L-form had irregular morphology with larger size than normal *S. aureus* and contained a large number of vesicles. The L-form bacteria had deficient or fractured cell wall. (D). Normal *S. aureus* with spherical shape and thick cell wall structure (TEM, ×100,000). (E). *S. aureus* L-form (TEM, × 10,000) showing polymorphic L-form bacteria with varying sizes and shapes. (F). *S. aureus* normal classical form (TEM, × 10,000) showing regular spherical morphology with homogeneous size and shapes. (G). *S. aureus* L-form colony (SEM, × 400). *S. aureus* L-form colony exhibited typical “fried egg” morphology. (H). *S. aureus* L-form colony inner structure (SEM, × 5000). In the inside of *S. aureus* L-form colony, the bacteria exhibited polymorphic shapes of varying sizes with the L-form colony structure showing similarity to a multilayered biofilm structure. The polymorphic bacteria are connected with large amounts of extracellular matrix materials (exopolysaccharide (EPS)) and lysed bacteria (arrow).

L-form induction media (LIM) was tested for its ability to induce *S. aureus* (Newman) to grow as L-form colonies. Stationary phase cells of *S. aureus* Newman strain produced L-form colonies when plated directly onto LIM. The minimum bacterial inoculum required for L-form colony formation was approximately 10^6^–10^7^ bacteria (the maximum dilution for 10^8^–10^9^ *S. aureus* bacteria to form L-form colony was 1∶100). This frequency is significantly lower than the L-form formation frequency of *E. coli* which is 10^4^–10^5^ [7]. The typical *S. aureus* L-form colonies had “fried egg” morphology (Fig. 1A) under inverted microscope in contrast to smooth colony of the normal form of *S. aureus* (Fig. 1B). The “fried-egg” L-form colonies had typical embedded growth into the soft agar and could not be scraped off in contrast to the normal classical forms which did not show embedded growth and could be scraped off easily from agar surface. Transmission electron microscopy (TEM) indicated that the *S. aureus* L-form bacteria had complete or partial loss of cell wall and contained a large number of intracellular vesicles (Fig. 1C) in contrast to the normal forms with cell wall without obvious vesicles (Fig. 1D). *S. aureus* L-form (TEM × 10,000) showed polymorphic sizes and shapes (Fig. 1E) in contrast to normal classical form showing morphology with homogeneous size and round shape with clear and smooth cell boundary (Fig. 1F). Scanning electron microscopy (SEM) indicated that *S. aureus* L-form colony exhibited rough surface morphology (Fig. 1G) while in the inside of the L-form colony the bacterial cells exhibited polymorphic morphologies of varying sizes (Fig. 1H) with the L-form colony structure showing similarity to biofilm structure. This is consistent with previous observation that L-form bacteria secrete exopolysaccharide (EPS) to the surface to prevent desiccation similar to biofilms and that defect in genes involved in EPS synthesis can cause lack of L-form growth [7].

### Screening for mutants with defect in L-form formation from *S. aureus* transposon mutant library

Having established the *S. aureus* L-form conditions, we wanted to identify genes that are involved in L-form formation. To do this, we first constructed a *S. aureus* transposon mutant library and then grew the library at 37°C overnight in 96-well plates followed by transfer of the mutants onto LIM plates as described in the Methods. Plates were incubated at 33°C for 7 days when mutants were scored for defect in forming L-form colonies.

**Figure 2:**
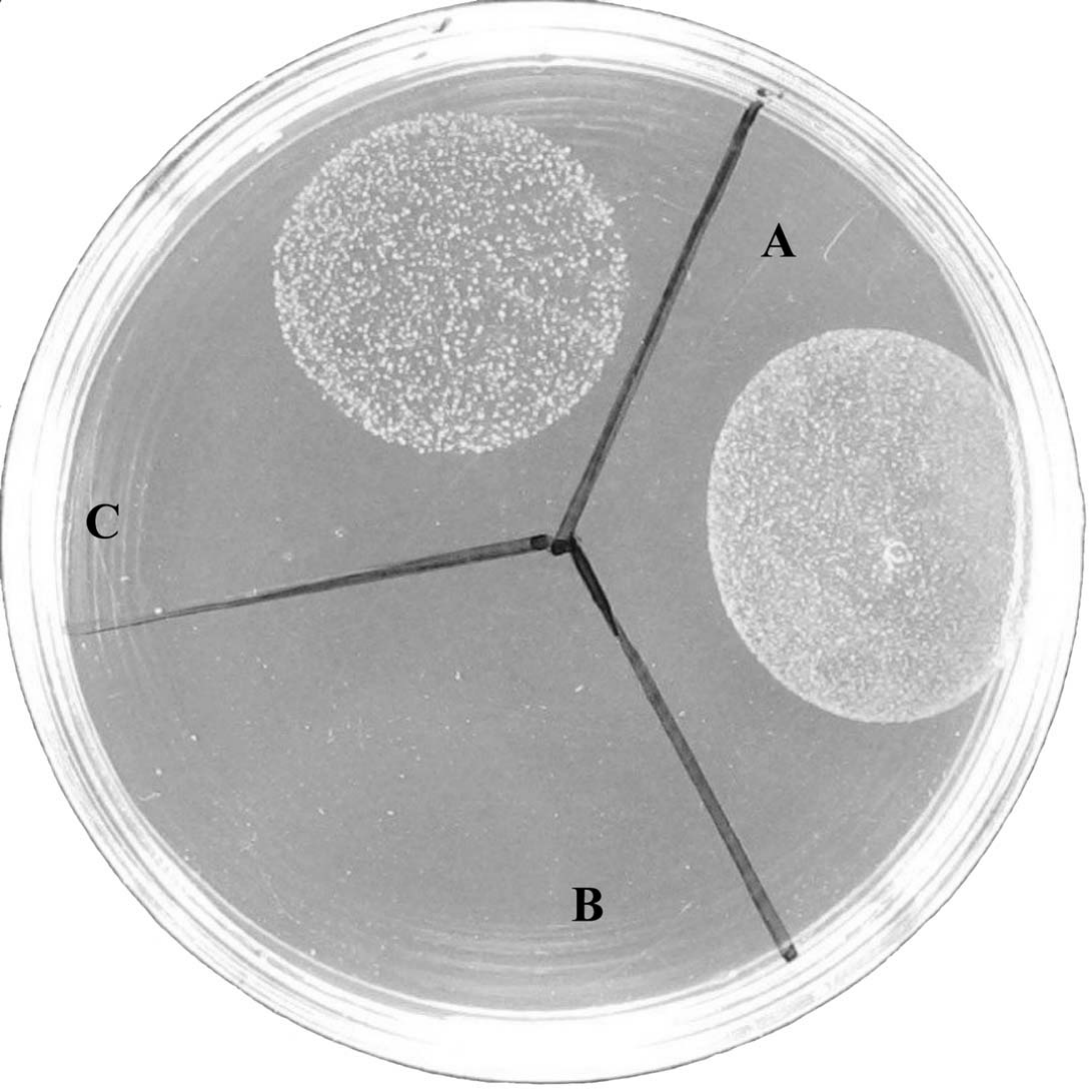
Results of *S. aureus* Newman, *glpF* mutant and the complemented strain in L-form formation. Stationary phase cultures of (A) *S. aureus* Newman, (B) *glpF* mutant-pT181 vector control, and (C) *glpF-*pT181-*glpF* complemented strain were spotted on L-form induction media (LIM) followed by incubation at 33°C for 5 days when the L-form growth was examined.

To identify the genes whose mutation led to defect in L-form formation, we performed inverse PCR as described in the Methods. Using inverse PCR and DNA sequencing we were able to identify 12 genes from 15 mutants, 3 of which mapped to *glpF* and *NWMN_0623* each, 1 mapped to *glpK*, 1 mapped to gluconate kinase (*gntK*), *NWMN_0623*, *NWMN_0872* (GTP pyrophosphokinase), *NWMN_1269* (sodium:alanine symporter family protein), 2 mapped to hypothetical proteins *NWMN_0333* and *NWMN_0843* of unknown function, 3 mapped to intergenic region. Because we found 4 mutants (3 in *glpF* and 1 in *glpK*) mapped to glycerol metabolism genes which predominate among these identified genes, we therefore focused and further characterized the role of glycerol metabolism genes in this study. To determine if the mutated *glpF* is indeed responsible for defective L-form formation, we attempted to complement the *glpF* mutant with the wild type *glpF* gene using the plasmid vector pT181. However, the initial attempt was unsuccessful when the complemented *glpF* mutant was plated directly on LIM plates. Since our previous work with *E. coli* L-form complementation indicated that inducible expression of the gene involved in L-form formation is critical for successful complementation of L-form defect of the mutants [7], we therefore induced the complemented *glpF S. aureus* strain containing tetracycline inducible vector pT181 with tetracycline in liquid culture prior to plating on LIM plates. This led to successful complementation of the *glpF* mutant with the wild type *glpF* gene. However, the effect of the complementation was partial (Fig. 2C) compared with the parent strain Newman (Fig. 2A), while the *glpF* mutant transformed with the pT181 vector control did not form any L-form colonies (Fig. 2B).

### *glpF* and *glpK* were overexpressed in *S. aureus* L-form bacteria but not in normal bacteria

**Figure 3:**
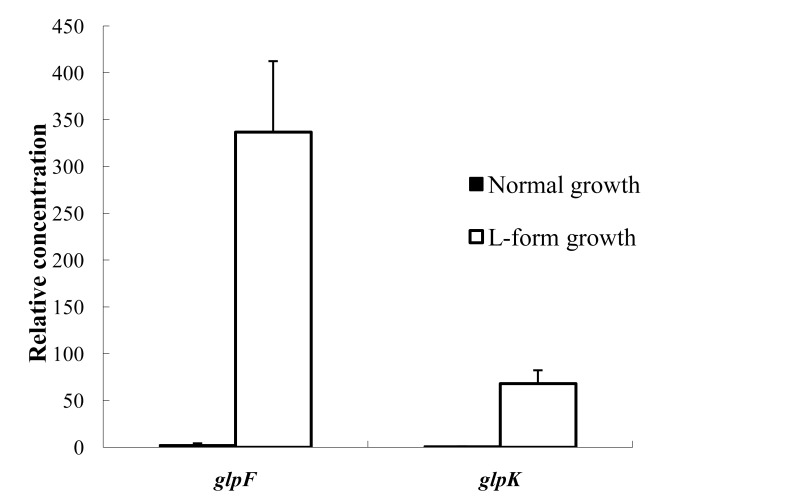
Relative concentrations of *glpF* and *glpK* expression in normal growth and L-form *S. aureus* as assessed by RT-PCR.

Since we identified mutations in *glpF* and *glpK* caused defect in L-form growth, we wanted to know if *glpF* and *glpK* are overexpressed in *S. aureus* L-form bacteria compared with normal classical form *S. aureus*. To confirm this, we prepared *S. aureus* L-form bacteria from L-form media LIM and normal *S. aureus* growth as a control on media without penicillin and isolated RNA from both types of the bacterial cells. The isolated RNA samples were then subjected to RT-PCR. *glpF* and *glpK* were found to be expressed at very low levels in normal growth but were significantly induced to 144-fold and 68-fold higher respectively in L-forms than in the normal control growth (*P<*0.05) (Fig. 3).

### Effect of glycerol on restoring L-form formation in *glpF* mutant

**Figure 4:**
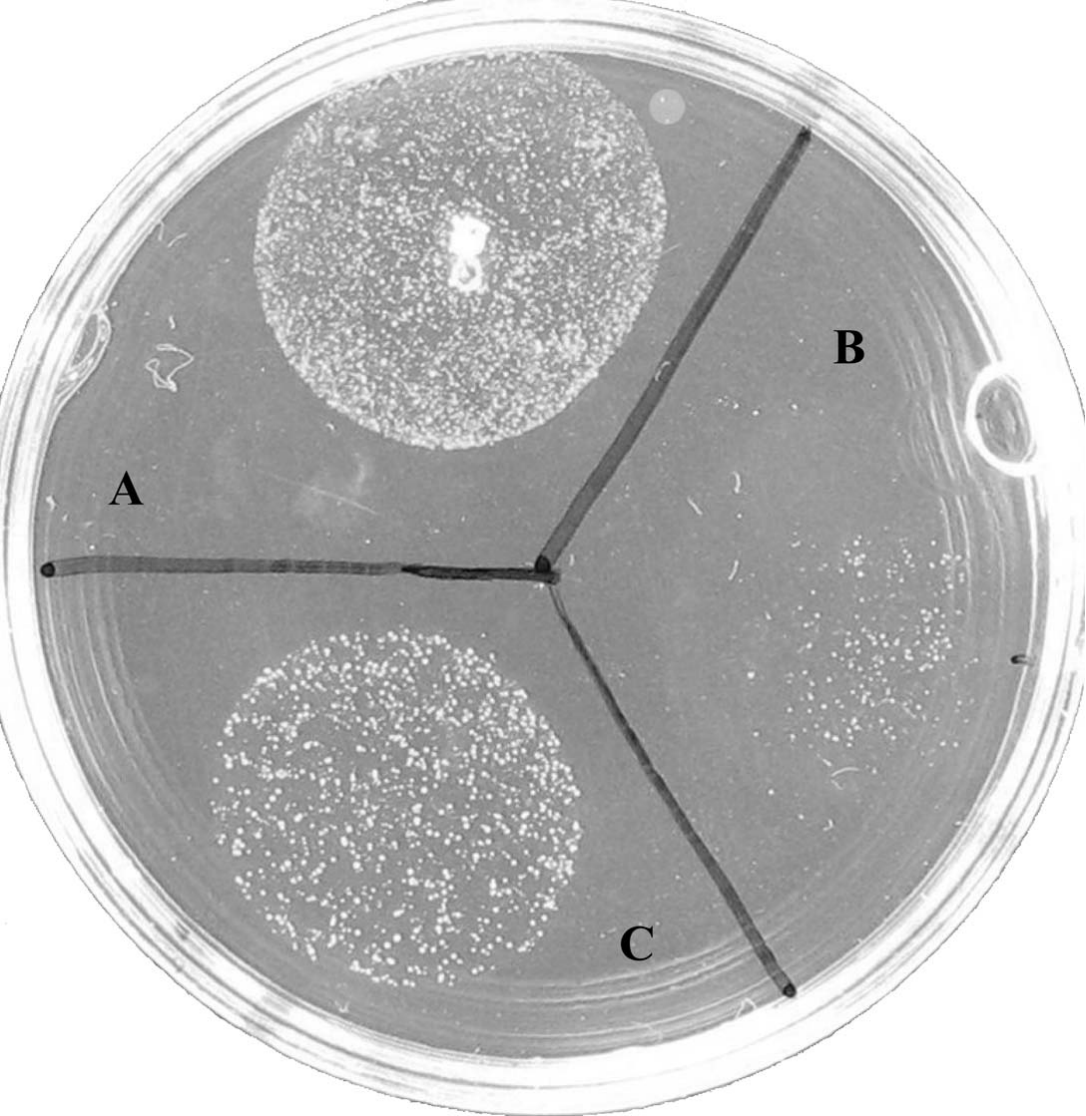
Effect of glycerol on L-form growth for *S. aureus* Newman, *glpF* mutant and the complemented strain. Stationary phase cultures of (A) *S. aureus* Newman, (B) *glpF* mutant-pT181 vector control, and (C) *glpF-*pT181-*glpF* complemented strain were spotted on L-form induction media (LIM) containing 1% glycerol followed by incubation at 33°C for 5 days when the L-form growth was assessed.

Since GlpF is involved in glycerol transport, we wanted to address the role of exogenously added glycerol in L-form formation in *S. aureus.* To do so, we incorporated varying concentrations of glycerol 0, 0.1%, 1% and 10% glycerol into the LIM. In LIM media with 0 and 0.1% glycerol, only wild type *S. aureus* Newman strain and the *glpF* complemented strain grew but *glpF* mutant failed to grow (Table 3). However, at 1% glycerol, the *glpF* mutant formed 1.03×10^4^ L-form colonies but its efficiency was much lower than the wild type (7.75×10^5^ L-form colonies) and the *glpF* complemented strain (2.90×10^5^ L-form colonies) (Fig. 4, Table 3). The 1% glycerol only marginally increased the number of L-form colonies of the wild type strain by about 2 fold (Table 3). In contrast, at 10% glycerol, none of the strains Newman, the *glpF* mutant, or the *glpF* complemented strain formed L-form colonies.

**Table 3:** Effect of glycerol on L-form formation of the *glpF* mutant.

	Wild type	*glpF* mutant	*glpF* complementation
Spotted bacterial number on TSA (cfu/ml)	3.63×10^9^	1.73×10^9^	5.77×10^9^
L-form colony number on LIM (cfu/ml)	3.1×10^5^	0	2.15×10^5^
L-form colony number on 1% glycerol LIM (cfu/ml)	7.75×10^5^	1.03×10^4^	2.90×10^5^

To address if the role of 1% glycerol is to serve as an osmoprotectant in facilitating L-form formation in the *glpF* mutant, we added known osmoprotectant NaCl at 4.5% to the LIM and assessed if it could allow the *glpF* mutant to grow as L-forms. However, supplementation of NaCl while allowing L-form formation in wild type and complemented *glpF* mutant, failed to facilitate L-form formation in the *glpF* mutant (data not shown).

### Defective persistence of *S. aureus glpF* mutant in antibiotic exposure assays

**Figure 5:**
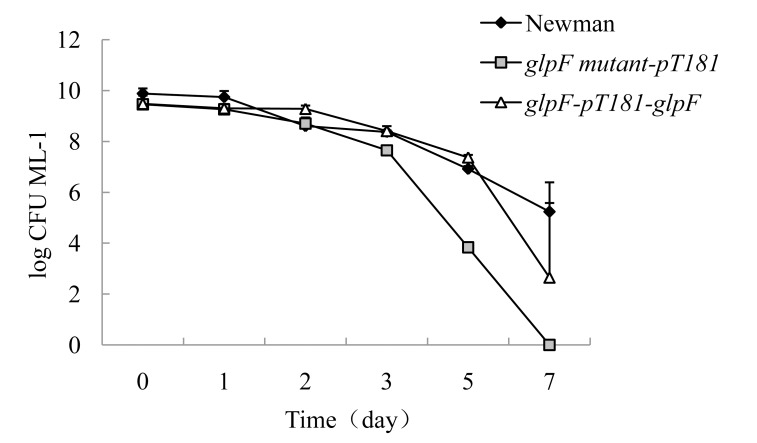
Survival of stationary phase cultures of the *S. aureus glpF* mutant transformed with vector pT181, or pT181+wild type *glpF*, and the parent strain upon ampicillin (50 µg/ml) exposure over time.

**Figure 6:**
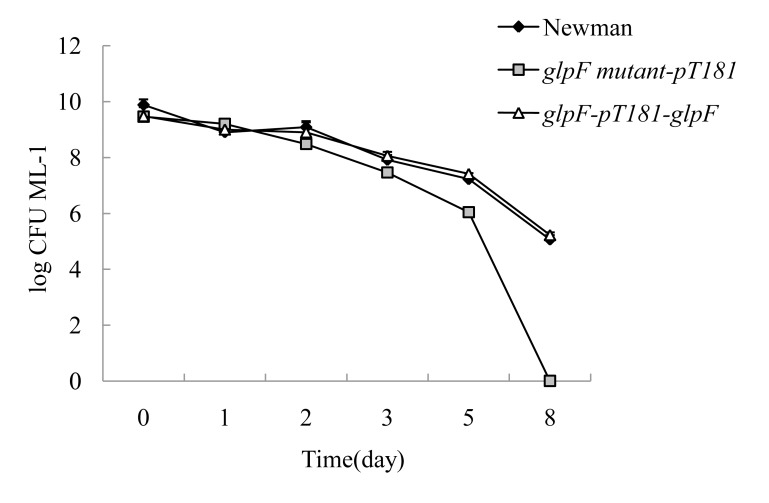
Survival of stationary phase cultures of the *glpF* mutant, the complemented strain, and the parent strain upon norfloxacin (40 µg/ml) exposure over time.

In our previous study with *E. coli* L-form bacteria, we found that genes involved in L-form formation overlapped with those involved in antibiotic persistence [7]. Exposure of the stationary phase culture of *glpF* mutant to ampicillin (50 µg/ml) revealed that the mutant began to show defect in persistence at day 3 but the defect was more obvious after 5 days. After 7 days, no CFU was detectable in the *glpF* mutant transformed with the vector control pT181; in contrast, the complemented strain (transformed with pT181+*glpF*) and the parent strain Newman had 100–1000 and 10,000 CFUs remaining (Fig. 5) (Table 4). For exposure to norfloxacin (40 µg/ml), the *glpF* mutant had a similar trend as ampicillin treatment, where it reached zero CFU by day 8. Complementation of the *glpF* mutant with the wild type *glpF* gene only partially restored the persister levels compared with parent strain for ampicillin exposure (Fig. 5) but caused full restoration of persister levels for norfloxacin exposure (Fig. 6) (Table 4).

**Table 4:** Survival of stationary phase cultures of the *glpF* mutant, the complemented strain and the parent strain upon antibiotic exposure over time.

Antibiotics	Time	No. of bacteria (log CFU ML^−1^, mean ±SD)		
		Newman	*glpF* mutant-pT181	*glpF*-pT181-*glpF*
Ampicillin	0	9.88±0.20	9.46±0.11	9.48±0.32
(50 µg/ml)	1d	9.74±0.24	9.26±0.21	9.30±0.17
	2d	8.60±0.09	8.70±0.24	9.28±0.13
	3d	8.37±0.23	7.65±0.07	8.41±0.02
	5d	6.92±0.11	3.84±0.09	7.37±0.10
	7d	5.24±0.34	0	2.65±3.74
Norfloxacin	0	9.88±0.20	9.46±0.11	9.48±0.16
(40 µg/ml)	1d	8.90±0.09	9.2±0.13	9.0±0.17
	2d	9.08±0.22	8.48±0.11	8.90±0.36
	3d	7.91±0.29	7.46±0.04	8.06±0.03
	5d	7.22±0.02	6.04±0.01	7.41±0.03
	8d	5.06±0.16	0	5.22±0.10

**Figure 7:**
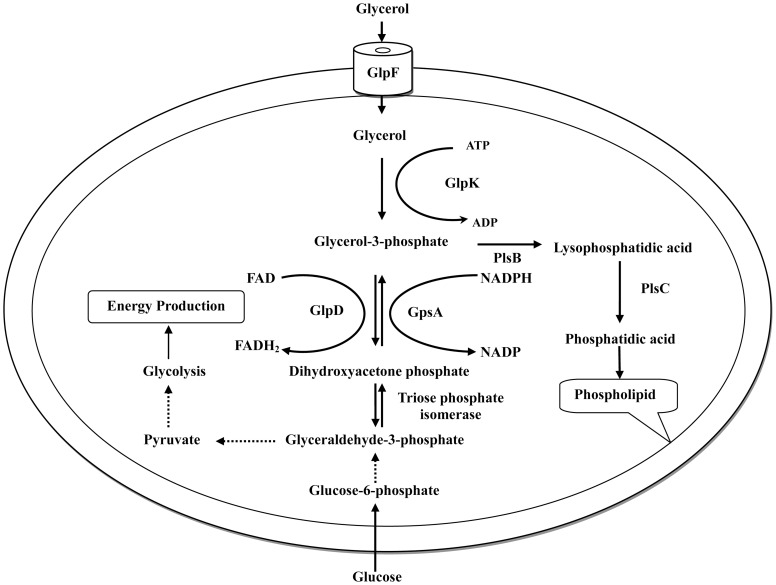
Glycerol uptake and metabolic pathway. GlpF is involved in uptake of glycerol into *S. aureus* while GlpK is glycerol kinase and is involved in converting glycerol to glycerol-3-phosphate which then can be used for synthesis of cell membrane phospholipid and also for energy production.

Although many previous studies have demonstrated the formation of L-forms by *S. aureus* [13], [14], [17], the mechanisms involved in L-form formation has remained unknown in this organism. This study provided the first molecular insight into the mechanism of L-form formation in *S. aureus* by demonstrating the important role of glycerol uptake in the L-form formation. GlpF is involved in glycerol uptake (Fig. 7), and the observation that *glpF* mutation causes defect in L-form growth in *S. aureus* suggests uptake of glycerol is essential for L-form formation. The mechanism by which GlpF is involved in L-form formation in *S. aureus* is likely mediated through its role in production of energy via pyruvate entry into TCA cycle and glycolysis or alternatively through cell membrane synthesis via lysophosphatidic acid (LPA) to phospholipids (Fig. 7) to strengthen membrane integrity required for L-form growth in the absence of cell wall. This study represents the first effort at identifying the mechanisms of L-form formation in *S. aureus,* and further studies are needed to explore other possible mechanisms of L-form formation besides glycerol uptake in future studies.

It is interesting to note that supplementation of appropriate concentration of glycerol (1%) partially compensated for the loss of L-form formation to *glpF* mutant (Fig. 4). GlpF is known to be a member of the aquaporin (AQP) channel family. Some of AQPs channels that conduct water and also conduct glycerol (aquaglyceroporins) [22]. When the environmental glycerol is increased, the aquaporin (AQP) channel may partially compensate for the loss of GlpF and help the *glpF* mutant to form a small number of L-form colonies (Fig. 4). We believe the role of glycerol in L-form formation is mediated through its uptake and metabolism rather than an osmoprotectant role in L-form formation for the following reasons. First, we demonstrated in the new experiment that adding 4.5% NaCl as an osmoprotectant did not help the *glpF* mutant to form L-forms while it allowed wild type and the complemented *glpF* mutant strains to form L-form colonies. This indicates the defect in *glpF* mutant cannot be complemented by other osmoprotectant like NaCl. Second, if the role of glycerol were to serve as osmoprotectant, we would expect that higher concentration of glycerol would facilitate L-form formation. However, we found higher glycerol content, e.g., 10% glycerol did not allow L-forms to form. Third, our finding that mutation in *glpK* which is glycerol kinase involved in glycerol metabolism caused defect in L-form formation also does not support glycerol serving as an osmoprotectant in facilitating L-form formation. This is because GlpK is glycerol kinase that converts glycerol to glycerol-3-phosphate which can be used for synthesis of cell membrane phospholipid and also for energy production to facilitate L-form formation. However, high glycerol concentration (10%) inhibited the L-form growth for wild type as well as the mutant *S. aureus*, presumably because high concentrations of glycerol produce toxic metabolites thus preventing L-form growth.

It is noteworthy that in addition to its role in L-form formation, *glpF* is also involved in tolerance or persistence to antibiotics in *S. aureus* as demonstrated by a defect in persistence in *glpF* mutant upon exposure to ampicillin or norfloxacin (Fig. 5 and Fig. 6). This finding is consistent with the previous observation that genes involved in glycerol metabolism such as *glpD* encoding sn-glycerol-3-phosphate dehydrogenase and *plsB* encoding sn-glycerol-3-phosphate acyltransferase (Fig. 7), have been found to be involved in persister formation [23]. Our findings that glycerol uptake is important for both L-form and persistence of *S. aureus* and provide further support for the close relationship between the two entities as has been observed for *E. coli* [7]. This finding is consistent with the recent proposal that L-form bacteria are related to persisters and are part of the heterogeneous persister continuum [24]. The only difference is that frequency of L-form forming bacteria is 2–3 orders of magnitude lower than persister frequency [24] and can be considered “deep” persisters [25]. Although previous study with *E. coli* L-form bacteria did not identify *glpF* being critical for L-form formation as in *S. aureus*, microarray analysis indicated that glycerol metabolism gene *glpD* encoding sn-glycerol-3-phosphate dehydrogenase is upregulated in *E. coli* L-form bacteria [7], suggesting glycerol metabolism could be important for L-form bacteria in different bacterial species.

In conclusion, we established an L-form model in *S. aureus*, characterized their morphologies and optima conditions of their formation, and identified genes involved in glycerol uptake and metabolism being important for L-form formation and persistence in *S. aureus.* These findings shed new light on the mechanisms of L-form formation and persister biology in *S. aureus* and may have implications for development of new drugs targeting persisters for improved treatment of persistent bacterial infections.
